# Supplementary figures and images for: Not All That Is Gold Glitters: PV-IRES-Cre Mouse Line Shows Low Efficiency of Labeling of Parvalbumin Interneurons in the Perirhinal Cortex
Source: Front Neural Circuits. 2021 Nov 8;15:781928. doi: 10.3389/fncir.2021.781928 (PMC8606682; doi:10.3389/fncir.2021.781928)

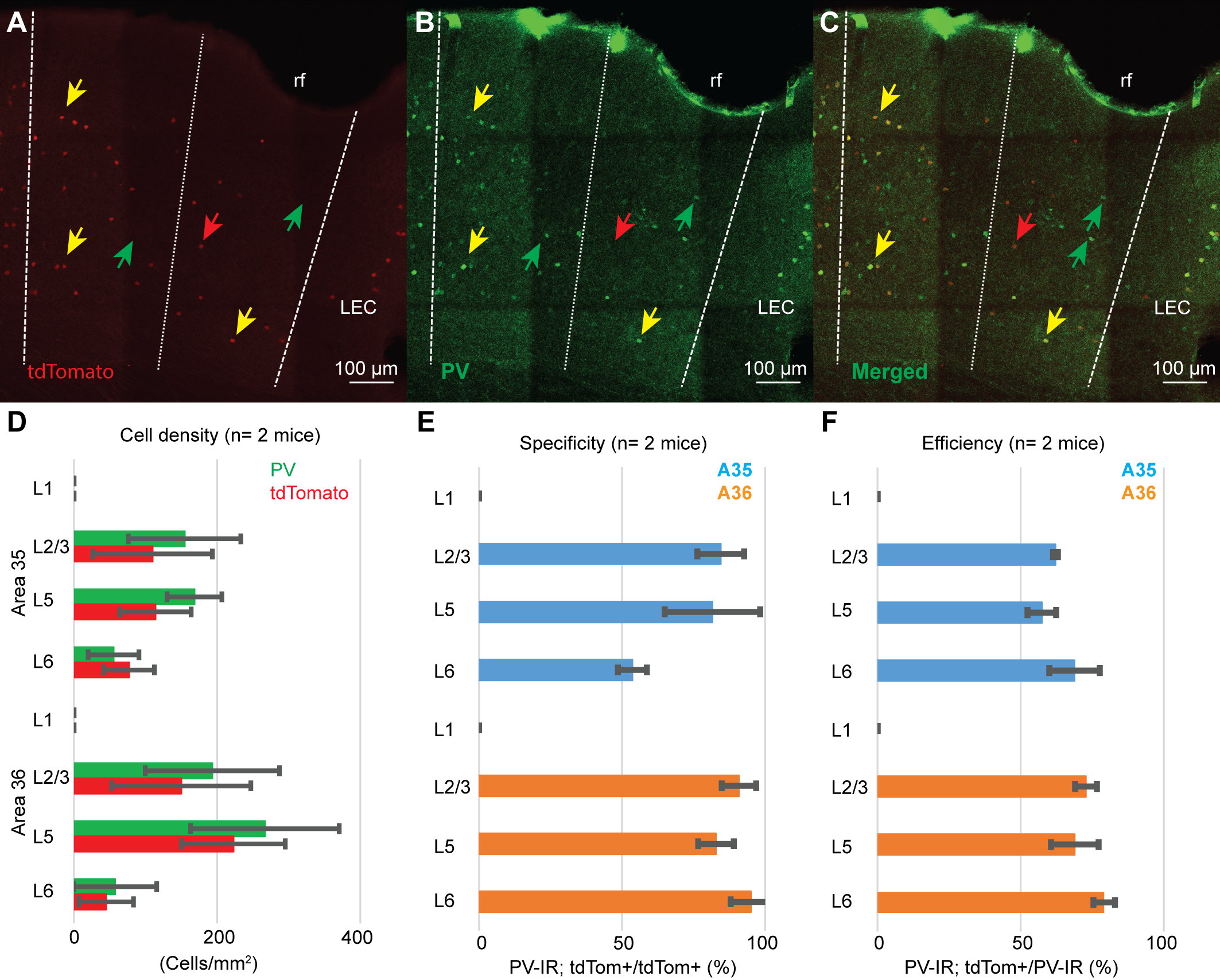

Supplement: Supplementary Figure 1 — (A–C) Representative immunostainings showing the expression of tdTomato (A, red), PV (B, green) and the merged image (C) in the perirhinal cortex of the PV-IRES-Cre line using a primary mouse anti-PV antibody. (D) Bar plot showing the densities of PV immunoreactive (PV-IR) neurons (green) and tdTomato expressing neurons (red). (E) Bar plot showing the specificity obtained with the mouse anti-PV. (F) Efficiency obtained with the mouse anti-PV. © 2011 Allen Institute for Brain Science. Allen Mouse Brain Connectivity Atlas. Available from: https://connectivity.brain-map.org/. [file Image_1.TIF]

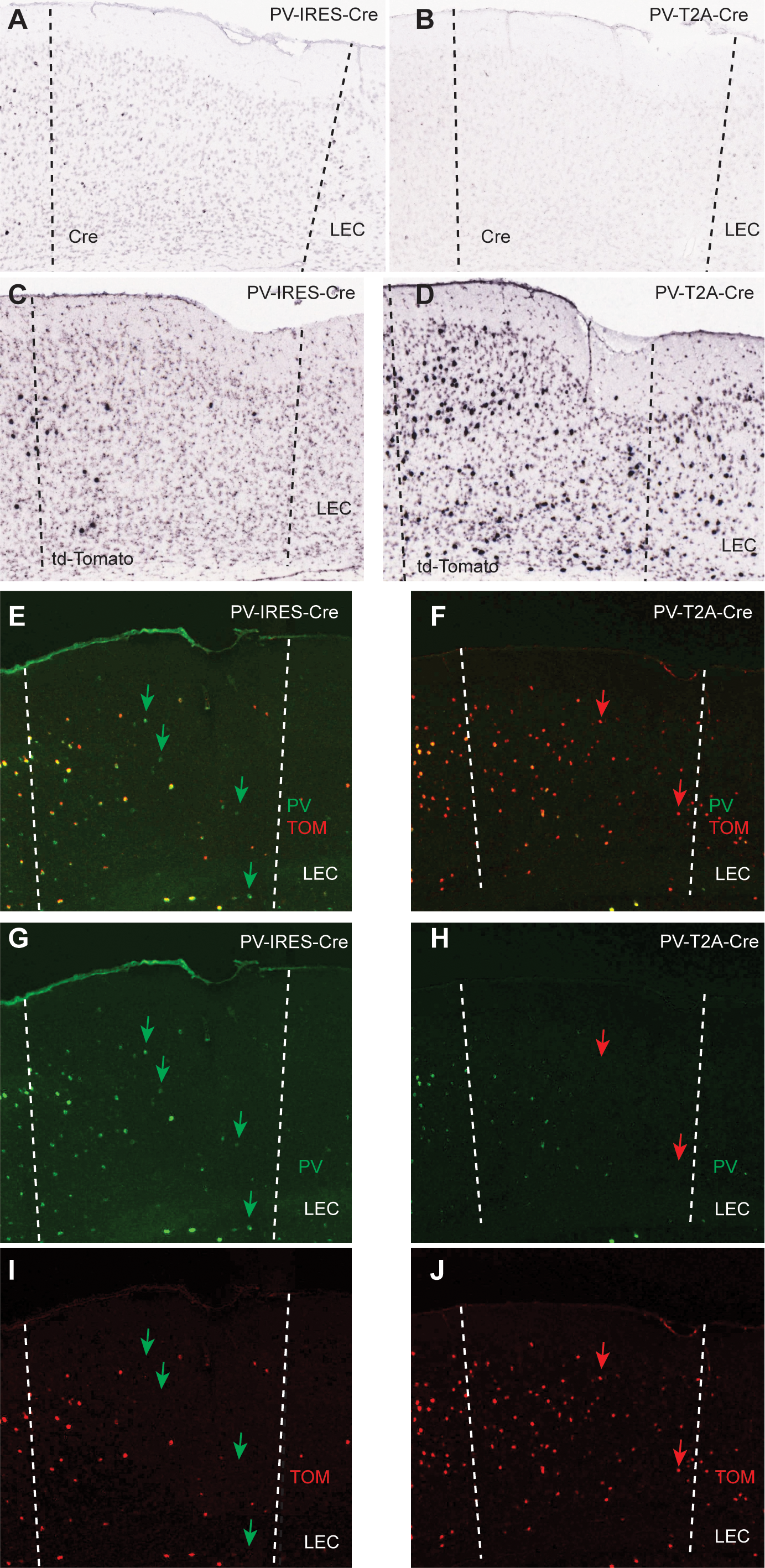

Supplement: Supplementary Figure 2 — Comparison of the PV-IRES-Cre and PV-T2A-Cre mouse lines in the PER. (A) PER of a PV-IRES-Cre mouse showing ISH for Cre. (B) PER of a PV-T2A-Cre mouse showing ISH for Cre. (C) PER of a PV-IRES-Cre mouse showing ISH for td-Tomato. (D) PER of a PV-T2A-Cre mouse showing ISH for tdTomato. (E–J) FISH for PV and td-Tomato in PV-IRES-Cre (E, merged; G, PV; I, td-Tomato), and in PV-T2A-Cre (F, merged; H, PV; J, td-Tomato). Green arrows point to PV-expressing neurons that do not express tdTomato. Red arrows point to td-Tomato neurons that do not express PV. LEC, lateral entorhinal cortex. [file Image_2.TIF]
